# Hypertension Prevalence Based on Three Separate Visits and Its Association With Obesity Among Chinese Children and Adolescents

**DOI:** 10.3389/fped.2019.00307

**Published:** 2019-07-24

**Authors:** Qian Zhang, Lili Yang, Yanqing Zhang, Min Zhao, Yajun Liang, Bo Xi

**Affiliations:** ^1^Zibo Center for Disease Control and Prevention, Zibo, China; ^2^Department of Epidemiology, School of Public Health, Shandong University, Jinan, China; ^3^Department of Nutrition, School of Public Health, Shandong University, Jinan, China; ^4^Department of Public Health Sciences, Karolinska Institutet, Stockholm, Sweden

**Keywords:** blood pressure, hypertension, obesity, children, cross-sectional, repeated measurement

## Abstract

**Background:** Clinical practice guidelines recommended that hypertension in children and adolescents should be defined based on elevated blood pressure (BP) on at least three separate occasions. Therefore, in the present study, we aimed to estimate the prevalence of hypertension based on three separate visits among Chinese children and adolescents and to examine its relationship with obesity.

**Methods:** A school-based cross-sectional survey was performed in children and adolescents in Jinan, China between September 2012 and September 2014. A total of 7,832 children and adolescents aged 6–17 years were included. Anthropometric data and BP were measured by trained examiners. Elevated BP was defined as BP ≥ 95th percentile for age and sex based on the Chinese reference data. Participants with elevated BP at the first visit underwent a second visit 2 weeks later, and a third visit was conducted if BP was still high at the second visit. Hypertension was defined as having an elevated BP at all three visits. Obesity was defined in three ways by using body mass index, waist circumference, and waist-to-height ratio.

**Results:** The prevalence of elevated BP decreased substantially across three separate visits, with the prevalence of 17.2, 8.6, and 4.9%, respectively. Obesity was an independent risk factor for elevated BP during each visit. Based on the body mass index, obesity was associated with higher risk of elevated BP, with the adjusted odds ratios (ORs) and 95% confidence intervals (CIs) of 8.6 (6.8–11.0), 12.5 (9.1–17.3), and 14.0 (8.9–22.2), respectively, at the first, second and third visit. The ORs of elevated BP were similar in association with obesity defined by waist circumference or waist-to-height ratio.

**Conclusions:** The prevalence of hypertension based on three visits was ~5% in Chinese children and adolescents. There was a dose-response relationship between obesity and elevated BP across three visits.

## Introduction

Hypertension in adults has been one of the major contributors to cardiovascular disease burden worldwide. It is estimated that nearly 10.7 million deaths and 211.8 million disability adjusted life-years were due to hypertension worldwide in 2015 (1). Notably, pediatric elevated blood pressure (BP) tends to track into adulthood (2) and it is associated with a series of early target organ damages in childhood (3), which can increase the risk of cardiovascular diseases later in life. Therefore, recognition and control of elevated BP at an early age may be an important strategy for reducing the hypertension-induced cardiovascular disease burden.

BP in children may vary a lot due to several potential reasons, such as the “white coat effects” and environmental factors (e.g., temperature and noise), which may cause transient elevated BP. Therefore, hypertension in children defined based on the BP measured at one single visit may overestimate the true prevalence. Indeed, clinical practice guidelines recommended that hypertension in children and adolescents should be defined based on elevated BP on at least three separate occasions (4–6). Up to now, several studies from western pediatric populations have assessed the prevalence of hypertension based on this recommendation (7, 8). However, few are from Chinese children and adolescents (9, 10). In addition, different populations may have different growth and development patterns and different exposures to lifestyle factors.

Adiposity has been shown to be an independent risk factor for elevated BP in children. However, most analyses were conducted using body mass index (BMI) as the indicator to assess weight status, which cannot distinguish fat distribution. Waist circumference (WC) and waist-to-height ratio (WHtR) are indicators of abdominal obesity that can better assess the distribution of the visceral adipose tissue (11).

Thus, based on a school-based, cross-sectional survey conducted in Jinan, China, we aimed to estimate the true prevalence of hypertension among Chinese children and adolescents aged 6–17 years. In addition, we examined the relationship between elevated BP and excess weight defined in three ways.

## Materials and Methods

### Study Population

This study was conducted in four schools in urban region of Jinan, China between September 2012 and September 2014. The four public schools, including two primary schools, one junior high school and one senior high school, were chosen using convenient cluster sampling method. All students from the selected schools were invited to participate in the survey. A standard questionnaire, including demographic information, family history of hypertension, puberty status, and lifestyle factors, was finished by the students and/or their parents. Physical examinations (i.e., height, weight, WC, and BP) were conducted by trained research staff according to a standard protocol. A total of 7,832 students were included in the present study after excluding those with missing information on age, sex, height, weight, WC, and BP. Signed informed consent was obtained from all students and their guardians. Ethical approval was obtained from the Ethics Committee of the Capital Institute of Pediatrics in Beijing, China.

### BP Measurements and Definitions

BP was measured by trained examiners with a clinically validated electronic device (OMRON HEM-7012) (12). After at least 10 min of rest, BP was measured on the right arm supported at the level of heart, in a sitting position with the back supported and feet flat on the floor. The mid-arm circumference was measured and appropriate cuff size was chosen (i.e. small, normal, or large cuff for a mid-arm circumference of 13.0–21.9, 22.0–31.9, or 32.0–42.0 cm, respectively). Three consecutive BP measurements were taken with at least 1 min apart. If the difference between any two of the three BP readings was more than 5 mmHg, then a fourth BP measurement was conducted. Mean value of the last two readings was used for data analysis.

**Figure 1:**
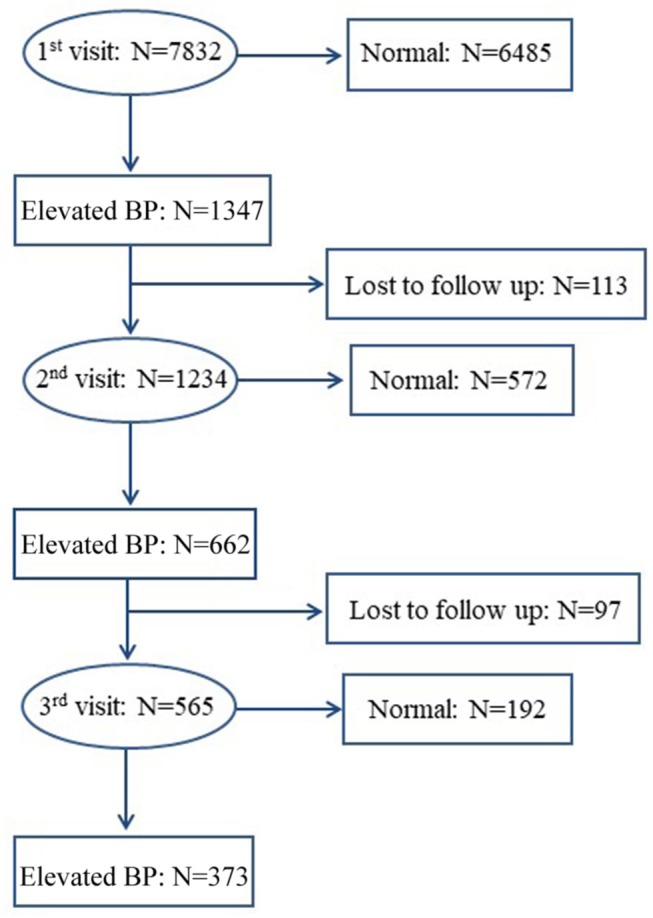
The flow chart of blood pressure screening procedures.

Elevated BP was defined if systolic BP (SBP) and/or diastolic BP (DBP) ≥age- and sex-specific 95th percentiles of the Chinese BP references (13). Participants with elevated BP at the first visit underwent a second visit at least 2 weeks later. If elevated BP persisted at the second visit, a third visit was conducted at least another 2 weeks later, according to the same procedures. Those with elevated BP at all the three visits were identified as hypertensive (4–6). The flow chart of the BP screening procedures is shown in Figure 1. One hundred thirteen of 1,347 children and adolescents with elevated BP at the first visit and 97 of 662 children and adolescents with sustained elevated BP at the second visit were lost to follow-up for some reasons, such as refusal, absence from school, or having a disease. We compared the baseline characteristics between followed-up participants and those lost to follow-up and we found no significant differences (data not shown).

### Adiposity Measurements and Definitions

Weight was measured twice in light clothes and without shoes using an electronic scale, and was approximated to 0.1 kg. Height was also measured twice and approximated to 0.1 cm. The mean of two measures was used for analysis. BMI was calculated as the weight divided by the height squared (kg/m^2^). Normal weight, overweight, and obesity were defined based on the age- and sex-specific BMI cutoffs for Chinese children and adolescents (14). WC was also measured twice and approximated to 0.1 cm using a standard tape. Extend the tape around the waist in a horizontal plane at the level of 1 cm above the umbilicus. Central overweight and obesity were defined according to the age- and sex-specific WC cutoffs of Chinese children and adolescents (15). WHtR was calculated as the ratio of WC and height. WHtR ≥0.50 was used to define central obesity (16).

### Covariates

The collected information includes age, sex, birth weight, puberty status, parental history of hypertension, and parental education levels. Puberty status was dichotomized based on whether they had menarche for girls or spermatorrhoea for boys. Parental history of hypertension (yes vs. no) was obtained from both father and mother using the following question: Have you ever been told by a physician to be hypertensive or taking anti-hypertensive drugs currently? Parental education levels were categorized into primary school, high school, college or university.

### Statistical Analysis

Data were analyzed with SAS, version 9.3 (SAS Institute, Cary, NC, USA). Quantitative variables were presented as mean ± standard deviation (or standard error). The differences in quantitative variables between groups were compared by covariance analysis adjusted for age and sex (or BMI), and linear regression analysis was used to test trends. Categorical variables were expressed as percentages and the differences between groups were compared by chi-square test and Cochran-Armitage analysis was used for trend tests. Binary logistic regression analysis was used to assess the relationship between adiposity measures and hypertension, with adjustment of potential covariates. Odds ratios (ORs) with the corresponding 95% confidence intervals (CIs) were calculated. Two-sided *p* < 0.05 was considered as statistically significant.

## Results

### Characteristics of the Participants Across Three Different Visits

A total of 7,832 students (boys: 52.0%), aged 6–17 years, were included at the first visit, 1,234 (boys: 61.7%) at the second visit, and 565 (boys: 68.5%) at the third visit. Characteristics of the participants across the three separate visits are presented in Table 1. Boys had higher height, weight, BMI, WC, WHtR, and higher prevalence of general and central obesity than girls at each visit (all *p* < 0.001). Of note, the prevalence of obesity was particularly high in both genders at the third visit, with the estimates reaching 59.7, 66.6, and 55.0%, respectively, based on different adiposity measures including BMI, WC and WHtR.

**Table 1:** Characteristics of the participants across three different visits.

	**First visit**				**Second visit**				**Third visit**			
	**All**	**Boys**	**Girls**	***p***	**All**	**Boys**	**Girls**	***p***	**All**	**Boys**	**Girls**	***p***
***N***	7,832	4,076	3,756		1,234	761	473		565	387	178	
Age (years)	11.3 ± 0.1	11.3 ± 0.1	11.3 ± 0.1	0.404	12.2 ± 0.1	12.4 ± 0.1	11.8 ± 0.2	0.012	12.5 ± 0.1	12.6 ± 0.2	12.0 ± 0.3	0.044
Height (cm)	151.0 ± 0.1	153.1 ± 0.1	148.8 ± 0.1	<0.001	155.7 ± 0.2	158.7 ± 0.3	152.7 ± 0.4	<0.001	156.5 ± 0.4	159.4 ± 0.4	153.6 ± 0.6	<0.001
Weight (kg)	47.6 ± 0.1	50.8 ± 0.2	44.3 ± 0.2	<0.001	59.1 ± 0.5	64.4 ± 0.6	53.7 ± 0.7	<0.001	62.9 ± 0.7	68.1 ± 0.8	57.7 ± 1.2	<0.001
BMI (kg/m^2^)	20.1 ± 0.1	20.8 ± 0.1	19.4 ± 0.1	<0.001	23.5 ± 0.1	24.6 ± 0.2	22.3 ± 0.2	<0.001	24.8 ± 0.2	25.8 ± 0.3	23.7 ± 0.4	<0.001
WC (cm)	67.6 ± 0.1	70.7 ± 0.2	64.6 ± 0.2	<0.001	75.5 ± 0.4	80.0 ± 0.5	71.0 ± 0.6	<0.001	78.6 ± 0.6	83.1 ± 0.6	74.2 ± 0.9	<0.001
WHtR	0.4 ± 0.1	0.5 ± 0.1	0.4 ± 0.1	<0.001	0.5 ± 0.1	0.5 ± 0.1	0.5 ± 0.1	<0.001	0.5 ± 0.1	0.5 ± 0.1	0.5 ± 0.1	<0.001
**BMI categories,%**												
Normal weight	59.1	53.3	65.3	<0.001	28.7	23.3	37.4	<0.001	17.5	12.9	27.5	<0.001
Overweight	21.0	21.7	20.3		24.6	23.9	25.8		22.8	23.3	21.9	
Obesity	19.9	25.1	14.4		46.7	52.8	36.8		59.7	63.8	50.6	
**WC categories,%**												
Normal WC	52.3	48.4	56.5	<0.001	25.0	20.9	31.5	<0.001	16.1	12.7	23.6	0.003
Central overweight	19.9	19.4	20.4		19.0	18.3	20.1		17.4	17.1	18.0	
Central obesity	27.9	32.2	23.1		56.1	60.8	48.4		66.6	70.3	58.4	
**WHtR categories,%**												
Normal WHtR	80.9	73.7	88.8	<0.001	57.0	48.5	70.6	<0.001	45.0	38.8	58.4	<0.001
Central obesity	19.1	26.3	11.2		43.0	51.5	29.4		55.0	61.2	41.6	

*Data are shown as mean ± standard error*.

*Covariance analysis adjusted for sex and age if appropriate*.

### Prevalence of Elevated BP Across Three Different Visits

The levels of SBP and DBP increased greatly across the three separate visits, and the similar trends were found in the subgroups by sex and age (Table 2). Table 3 shows the prevalence of elevated BP across three separate occasions among Chinese children and adolescents by age and sex. The prevalence of elevated BP was 17.2, 8.6, and 4.9% at the first, second and third visit, respectively. There was a downward trend in the prevalence of elevated BP over three repeated visits (*p* for trend<0.001). There were similar trends in elevated SBP and DBP across three visits, with the prevalence being 15.3, 8.2, and 4.8%, respectively, for elevated SBP and 6.0, 2.5, and 1.2%, respectively, for elevated DBP.

**Table 2:** Mean blood pressure values across three different visits.

	**Visit**	**SBP, mmHg**	**DBP, mmHg**
Total	First	109.5 ± 0.1	64.3 ± 0.1
	Second	122.3 ± 0.3	69.6 ± 0.2
	Third	126.4 ± 0.4	70.7 ± 0.4
	*p* for trend	<0.001	<0.001
Boys	First	111.6 ± 0.1	63.9 ± 0.1
	Second	125.1 ± 0.4	68.5 ± 0.3
	Third	129.3 ± 0.5	69.2 ± 0.4
	*p* for trend	<0.001	<0.001
Girls	First	107.5 ± 0.2	64.7 ± 0.1
	Second	119.6 ± 0.5	70.6 ± 0.4
	Third	123.6 ± 0.8	72.1 ± 0.6
	*p* for trend	<0.001	<0.001
6–11 years	First	107.2 ± 0.2	63.7 ± 0.1
	Second	118.7 ± 0.5	69.0 ± 0.4
	Third	119.9 ± 0.8	69.9 ± 0.6
	*p* for trend	<0.001	<0.001
12–17 years	First	111.8 ± 0.2	64.8 ± 0.1
	Second	125.7 ± 0.4	69.2 ± 0.3
	Third	130.4 ± 0.6	71.1 ± 0.5
	*p* for trend	<0.001	<0.001

*Data are shown as mean ± standard error*.

*Covariance analysis adjusted for sex, age, and BMI*.

**Table 3:** Prevalence of elevated blood pressure across three different visits by sex and age.

	**Visit**	**Elevated SBP**	**Elevated DBP**	**Elevated BP**
Total	First	15.3	6.0	17.2
	Second	8.2	2.5	8.6
	Third	4.8	1.2	4.9
	*p* for trend	<0.001	<0.001	<0.001
Boys	First	19.1	5.8	20.6
	Second	10.9	2.5	11.2
	Third	6.6	1.2	6.7
	*p* for trend	<0.001	<0.001	<0.001
Girls	First	11.2	6.1	13.6
	Second	5.2	2.4	5.8
	Third	2.8	1.3	2.9
	*p* for trend	<0.001	<0.001	<0.001
6-11 years	First	13.1	5.4	14.7
	Second	6.7	2.3	7.0
	Third	3.7	1.0	3.9
	*p* for trend	<0.001	<0.001	<0.001
12-17 years	First	17.4	6.5	19.6
	Second	9.5	2.6	10.1
	Third	5.8	1.4	5.9
	*p* for trend	<0.001	<0.001	<0.001

*Data are shown as percentages (%)*.

Elevated BP was significantly more prevalent in boys than girls during each visit (first: 20.6 vs. 13.6%, second: 11.2 vs. 5.8% and third: 6.7 vs. 2.9%). Elevated BP was also more prevalent in adolescents aged 12–17 years compared with children aged 6–11 years, with the prevalence of 19.6 vs.14.7%, 10.1 vs. 7.0%, and 5.9 vs. 3.9% at the first, second and third visit, respectively (Table 3).

### Associations Between Obesity and Elevated BP

Table 4 shows the associations of general and central obesity with elevated BP across three different visits. Obesity was an independent risk factor for elevated BP at each visit, irrespective of anthropometric indices used to define weight status. Based on the BMI definition, obesity was associated with higher risk of elevated BP, with the adjusted ORs (95% CIs) being 8.6 (6.8–11.0), 12.5 (9.1–17.3), and 14.0 (8.9–22.2), respectively, across the three visits. Corresponding values were 5.7 (4.6–7.2), 7.7 (5.6–10.6), and 10.9 (6.7–17.9), respectively, for the WC definition, and 4.9 (3.9–6.1), 6.3 (4.8–8.3), and 7.7 (5.3–11.1), respectively, for the WHtR definition.

**Table 4:** Associations of different adiposity measures with elevated blood pressure across three different visits.

		**First visit**		**Second visit**		**Third visit**	
		**OR (95%CI)**	***p***	**OR (95%CI)**	***p***	**OR (95%CI)**	***p***
BMI categories	Normal	1		1		1	
	Overweight	2.5 (1.9–3.2)	<0.001	2.5 (1.7–3.6)	<0.001	2.9 (1.7–5.1)	<0.001
	Obesity	8.6 (6.8–11.0)	<0.001	12.5 (9.1–17.3)	<0.001	14.0 (8.9–22.2)	<0.001
WC categories	Normal WC	1		1		1	
	Central overweight	1.8 (1.4–2.4)	<0.001	1.8 (1.2–2.8)	<0.001	2.6 (1.4–4.9)	<0.001
	Central obesity	5.7 (4.6–7.2)	<0.001	7.7 (5.6–10.6)	<0.001	10.9 (6.7–17.9)	<0.001
WHtR categories	Normal WHtR	1		1		1	
	Central obesity	4.9 (3.9–6.1)	<0.001	6.3 (4.8–8.3)	<0.001	7.7 (5.3–11.1)	<0.001

*Adjusted for age, sex, birth weight, puberty, family history of hypertension (father, mother), and parental education levels*.

## Discussion

Our study shows that the prevalence of pediatric elevated BP decreases substantially across three visits, from 17.2% at the first visit to 4.9% at the third visit. This trend might be explained by the “white-coat” effects (17, 18), or anxiety (19) that can result in increased BP levels. As children became more familiar with BP measurements from the first visit to the third visit, the BP measurements became more reliable. Thus, BP measured at the third visit might better reflect the true BP status of the children.

Previous studies have also reported substantial decreases in the prevalence of elevated BP in children across different visits. A study conducted in Switzerland showed that the prevalence of elevated BP was 11.4, 3.8, and 2.2%, respectively, at the first, second and third visit (8). Another study in Chinese pediatric population reported that the prevalence of elevated BP was more than halved between the first and third visit (18.2 vs. 3.1%) (9). A recent meta-analysis demonstrated that the prevalence of pediatric elevated BP decreased from 12.1% at the first visit to 2.7% at the third visit (7). The findings above are similar with our study. BP screening based on one occasional visit may mislabel a substantial number of children as hypertensive ones, which may lead to unnecessary stress for children/parents. Additionally, the Cardiovascular Risk in Young Study reported that repeated observations of elevated BP among children and adolescents enhanced the prediction of hypertension in adults (20). Altogether, these findings emphasized the necessity of BP measurements on at least three different occasions to estimate the true prevalence of hypertension in the pediatric population or to diagnose hypertension in clinical practice.

Boys had higher prevalence of elevated BP than girls in our study, which was similar to several previous studies (21, 22). Data from the Victorian Family Heart Study showed that estrogen receptors (ER)-alpha and ER-beta might play more important roles in the genetic regulation of BP in men, and the sex steroid-related genes may contribute to the observed sex differences in BP (23). In addition, boys were more likely to have adverse healthy behaviors [e.g., tobacco smoking (24), alcohol use (25) and sedentary behaviors (26)] than girls. Moreover, the prevalence of elevated BP was much higher in adolescents than in children, which was consistent with several previous studies (27, 28). Possible explanations for the age differences included intense hormonal changes and elevated insulin resistance during pubertal period, and a much higher prevalence of overweight and obesity in adolescents vs. children in the present study.

The study also presented that adiposity was an independent risk factor for elevated BP in children, similar with previous publications (8, 9, 29). The novelty of our study includes the use of different adiposity measures to define obesity. BMI is a widely used indicator of obesity, but it cannot distinguish fat distribution. WC and WHtR are indicators of abdominal obesity that can better assess the distribution of the visceral adipose tissue. The link between obesity and hypertension may be mediated partly by the hyperactivity of the sympathetic nervous system (SNS) (30, 31). In children, SNS can cause a hyperdynamic hemodynamic state (32), contributing to elevated BP. Obese children with elevated BP often have increased heart rate and BP variability, which also suggests a primary role of heightened SNS activity in the association between obesity and hypertension (33).

The major strengths of our study include the large sample size, a fair participation rate (first visit: 99.8%; second visit: 91.6%; third visit: 85.3%), and the well-standardized BP measurements. However, our study also has some limitations. First, BP re-assessments were only made in children who had elevated BP readings at the initial visit. Consequently, this may cause some underestimation in hypertension, especially when some children have normal BP at first visit, but may actually have an elevated BP (i.e., masked hypertension) at the subsequent visits. Second, we used the Chinese children and adolescents BP references (13) to define elevated BP across three visits, which made our results incomparable with others. Third, our data were collected using a convenient cluster sampling method in urban area of Jinan, China, thus, the generalizability of our results to the whole Chinese children might be limited. Finally, based on a cross-sectional study design, our study is unable to provide causal associations between adiposity and pediatric elevated BP, but reverse causation is unlikely.

## Conclusions

The current study demonstrates that the prevalence of elevated BP decreases substantially across three different visits, and the true prevalence of hypertension is about ~5% in Chinese children and adolescents. Our findings highlight the importance of repeated BP assessments over at least three visits before making a final diagnosis of pediatric hypertension. In addition, early accurate detection and effective control of hypertension in children should be emphasized, especially among those with excess body weight.

## Data Availability

The raw data supporting the conclusions of this manuscript will be made available by the authors, without undue reservation, to any qualified researcher.

## Ethics Statement

All subjects gave written informed consent in accordance with the Declaration of Helsinki. Signed informed consent was obtained from all students and their guardians. Ethical approval was obtained from the Ethics Committee of the Capital Institute of Pediatrics in Beijing, China.

## Author Contributions

QZ, LY, and BX conceptualized the study and drafted the article. QZ, LY, and YZ analyzed the data. YZ, MZ, YL, and BX revised the manuscript. All authors approved the final submitted and published versions.

### Conflict of Interest Statement

The authors declare that the research was conducted in the absence of any commercial or financial relationships that could be construed as a potential conflict of interest.

## Abbreviations

BMI: Body Mass Index
BP: Blood Pressure
CIs: Confidence Intervals
DBP: Diastolic Blood Pressure
ER: Estrogen Receptors
ORs: Odds Ratios
SBP: Systolic Blood Pressure
SNS: Sympathetic Nervous System
WC: Waist Circumference
WHtR: Waist-to-height Ratio.
